# Integrating movement ecology with biodiversity research - exploring new avenues to address spatiotemporal biodiversity dynamics

**DOI:** 10.1186/2051-3933-1-6

**Published:** 2013-08-05

**Authors:** Florian Jeltsch, Dries Bonte, Guy Pe’er, Björn Reineking, Peter Leimgruber, Niko Balkenhol, Boris Schröder, Carsten M Buchmann, Thomas Mueller, Niels Blaum, Damaris Zurell, Katrin Böhning-Gaese, Thorsten Wiegand, Jana A Eccard, Heribert Hofer, Jette Reeg, Ute Eggers, Silke Bauer

**Affiliations:** Department of Plant Ecology and Nature Conservation, Intitute of Biochemistry and Biology, University of Potsdam, Maulbeerallee 2, 14469 Potsdam, Germany; Berlin-Brandenburg Institute of Advanced Biodiversity Research (BBIB), Berlin, D-14195 Germany; Department of Biology, Ghent University, K.L. Ledeganckstraat 35, Gent, 9000 Belgium; Department of Conservation Biology, UFZ – Helmholtz Centre for Environmental Research, Permoserstr 15, Leipzig, 04318 Germany; Biogeographical Modelling, BayCEER, University of Bayreuth, Universitätsstr. 30, Bayreuth, 95447 Germany; Irstea, UR EMGR Écosystèmes Montagnards, 2 rue de la Papeterie-BP 76, St-Martin-d’Hères, F-38402 France; National Zoological Park, Smithsonian, Conservation Biology Institute, 1500 Remount Road, Front Royal, VA 22630 USA; Department of Forest Zoology and Forest Conservation, University of Göttingen, Buesgenweg 3, Göttingen, 37077 Germany; Landscape Ecology, Technische Universität München, Emil-Ramann-Str. 6, 85354 Freising-Weihenstephan, Germany; Environmental Systems Analysis, Institute of Geoecology, Technical University of Braunschweig, Langer Kamp 19c, Braunschweig, 38106 Germany; Department of Landscape Ecology, UFZ – Helmholtz Centre for Environmental Research, Permoserstr. 15, Leipzig, 04318 Germany; Department of Biology, University of Maryland, College Park, MD 20742 USA; Biodiversity and Climate Research Centre (BiK-F), Senckenberg Gesellschaft für Naturforschung, Senckenberganlage 25, Frankfurt (Main), 60325 Germany; Department of Biological Sciences, Goethe Universität, Max-von-Laue-Straße 9, Frankfurt (Main), 60438 Germany; Department of Ecological Modelling, Helmholz Centre for Environmental Research (UFZ), Permoserstr. 15, Leipzig, 04318 Germany; Department of Animal Ecology, Institute of Biochemistry and Biology, Universität Potsdam, Maulbeerallee 1, Potsdam, 14469 Germany; Department of Evolutionary Ecology, Leibniz Institute for Zoo and Wildlife Research (IZW) in the Forschungsverbund Berlin e.V., Alfred-Kowalke-Str. 17, Berlin, 10315 Germany; Department of Animal Ecology, Netherlands Institute of Ecology (NIOO-KNAW), P.O. Box 50, Wageningen, AB 6700 The Netherlands; Swiss Ornithological Institute, Seerose 1, Sempach, 6204 Switzerland

**Keywords:** Mobile links, Species coexistence, Community dynamics, Biodiversity conservation, Long distance movement, Landscape genetics, Individual based modeling

## Abstract

Movement of organisms is one of the key mechanisms shaping biodiversity, e.g. the distribution of genes, individuals and species in space and time. Recent technological and conceptual advances have improved our ability to assess the causes and consequences of individual movement, and led to the emergence of the new field of ‘movement ecology’. Here, we outline how movement ecology can contribute to the broad field of biodiversity research, i.e. the study of processes and patterns of life among and across different scales, from genes to ecosystems, and we propose a conceptual framework linking these hitherto largely separated fields of research. Our framework builds on the concept of movement ecology for individuals, and demonstrates its importance for linking individual organismal movement with biodiversity. First, organismal movements can provide ‘mobile links’ between habitats or ecosystems, thereby connecting resources, genes, and processes among otherwise separate locations. Understanding these mobile links and their impact on biodiversity will be facilitated by movement ecology, because mobile links can be created by different modes of movement (i.e., foraging, dispersal, migration) that relate to different spatiotemporal scales and have differential effects on biodiversity. Second, organismal movements can also mediate coexistence in communities, through ‘equalizing’ and ‘stabilizing’ mechanisms. This novel integrated framework provides a conceptual starting point for a better understanding of biodiversity dynamics in light of individual movement and space-use behavior across spatiotemporal scales. By illustrating this framework with examples, we argue that the integration of movement ecology and biodiversity research will also enhance our ability to conserve diversity at the genetic, species, and ecosystem levels.

## Introduction

Movement plays a pivotal role in shaping biodiversity patterns across spatiotemporal scales. It affects biodiversity directly and indirectly by determining patterns in species distribution and species interactions (e.g. [1, 2]) as well as patterns of changes in species’ traits and genetic diversity (e.g. [3–7]), or by modifying habitat structures and resource levels (e.g. [8–10]). Excellent examples of the close linkage between movement and biodiversity are provided by the numerous studies highlighting the importance of dispersal for species distributions (e.g. [11, 12]) and metapopulation dynamics (e.g. [13]), range shifts (e.g. [14–19]) and the linkage of (meta-)community dynamics (e.g. [20–23]). In particular, metacommunity theory acknowledges the importance of movement for the assembly of diverse communities, e.g. through dispersal resulting in either mass effects or species sorting [24]. Yet, the strong focus on the exchange of individuals between (sub-)populations entails the risk of overlooking the importance of other facets and types of movement and related ecological interactions.

**Figure 1:**
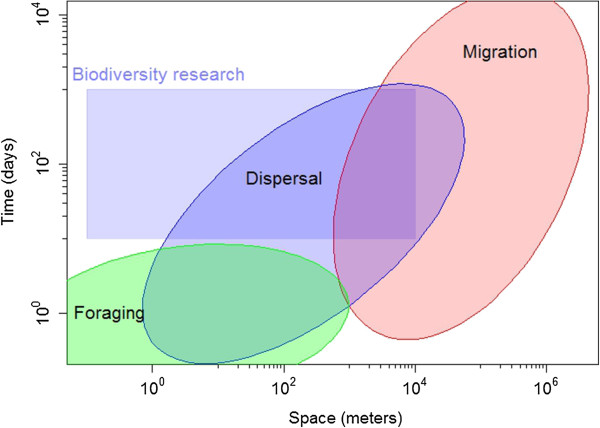
**A schematic overview of the typical spatial and temporal scales of different movement types of animals** **(including all body masses;** **a given species may cover only a small part of these spatio-** **temporal ranges):** **foraging** **(green ellipse),** **dispersal (** **blue ellipse)** **and migration (red ellipse).** These are also the typical scales at which studies investigate the fate of individuals or populations, which over large areas do not overlap with the scales at which biodiversity research typically takes place (grey rectangle).

Although many types of movements exist (e.g., movements to find a mate, defend a territory or nomadic movements), we here focus on three most common types of movements: foraging, dispersal, and migration [25]. While these different types of movements vary in many respects, the most striking difference lies in their spatiotemporal scales (Figure 1). Foraging movements are typically performed within a home range and several times per day, while dispersal refers to movements away from the place of birth towards another location or social environment for reproduction. Migratory movements often also track foraging conditions, but can easily cover several thousands of kilometers at once and the time required to complete migration may range from days to several months. In addition, foraging takes place repeatedly, more or less equally likely at every point in time throughout the year, while dispersal occurs at greater intervals and with peak times often occurring during specific seasons. Finally, migrations take place at regular intervals, e.g. spring and autumn migrations in seasonal environments.

Detecting the effect of these different movement types on biodiversity involves the inherent complexity of linking movement data to relevant ecological variables describing population and community performance and fitness, as well as species interactions. Problems of data insufficiency, as well as tractability of the underlying processes, propagate during upscaling of the movement process.

One of the major reasons why movement has been neglected in many biodiversity studies is the mismatch in the examined spatiotemporal scales (Figure 1). While biodiversity research typically focuses on species distributions or species coexistence, movement ecology deals with individuals and their interactions with one another and their (local) environments [26]. Both disciplines at best directly overlap in research oriented towards the (meta-)population level.

Given this mismatch of scales and research questions it is not surprising that movement aspects have until now only played a minor role in many biodiversity studies. However, establishing a more explicit link between movement ecology and biodiversity research could be a mutually stimulating endeavor, improving our understanding not only of ecological and evolutionary processes but also of applied aspects such as effective biodiversity conservation, for instance in light of biological invasions, climate change and landscape fragmentation.

The general need to learn more about organismal movement is reflected in the emergence and rapid expansion of ‘movement ecology’ as a new ecological discipline [26]. Technological advances now allow the acquisition of movement data in unprecedented quantity and quality, together with kinematic (e.g. acceleration), physiological (e.g. heart beat and temperature) and behavioral (e.g. vocalizations) information [26]. Such data fundamentally improve our understanding of the causes and consequences of individual movement and, in principle, open up new avenues to better integrate movement into biodiversity research. In this article we aim to provide a broader view and an initial suggestion for a conceptual framework for understanding movement effects on spatiotemporal biodiversity patterns. Based on a brief overview of how the three main movement types, i.e., foraging, dispersal and migration, impact biodiversity we develop the new integrated framework by merging the concept of movement ecology for individual organisms [26] with the concept of ‘mobile links’ [27] and an established theory on the maintenance of species diversity [28].

## Review

### Movement types and biodiversity

In the following, we provide a brief summary of the impact of the three main movement types on biodiversity at their respective scales.

#### Foraging movement

Foraging is fundamental to organisms’ survival and reproduction. It does not entail simply finding food but also includes many aspects from diet and habitat choice, functional response, trade-offs between food and safety, etc. (e.g. [29, 30]). Animals often forage relatively frequently and mostly within their home ranges. Although foraging has been investigated in great detail over the past decades, its implications for biodiversity dynamics have remained relatively unstudied. However, there are some exceptions: Edwards & Hollis [31] showed that latrines by cattle, ponies, and deer, i.e., movement and concentration of nutrients, affect both sward height and vegetation composition. Foraging movements of herbivores determine the spatiotemporal effect of grazing (and grazing heterogeneity) on plant communities [32–34] but also on invertebrates [35] and vertebrates [36]. Heterogeneous grazing and disturbance patterns can both increase and decrease diversity [37], but can also demonstrate the complexity of effects in different species groups with disturbance patterns also depending on predation risk to the foragers [38]. Howe [39] presented two contrasting seed deposition patterns during foraging movement (scatter vs. clump dispersal) which may have important consequences for plant performance. Scattering seeds may reduce predation, but plants growing in close vicinity and/or in clumps may have advantages in pollination [40]. Foraging behavior can also affect the distribution and fate of seeds in grasslands directly through endozoochory as well as change dispersal capacity and evolutionary dynamics [41].

#### Dispersal

Principally, the main drivers of dispersal are related to the avoidance of kin competition and inbreeding, bet hedging in spatiotemporal stochastic environments and escaping deteriorating environmental conditions [42]. In all cases, dispersal has a profound impact on the genetic structure of populations, either through gene flow when dispersal is successful, or by eroding the genetic variation in source populations when dispersal events lead to mortality and non-successful settlement. While gene flow is expected to be the main link-effect of successful dispersal movements, dispersal has also been demonstrated to be important in linking different populations with diseases [43], mutualistic endosymbionts [44], and nutrients through subsidies at the aquatic-terrestrial interface [45, 46] and along ecotones in agricultural landscapes [47]. Dispersal also links populations by impacting the level of synchronicity among populations, which in turn affects meta-population persistence [24]. Gene flow decreases the level of genetic differentiation among populations with migration load potentially destroying patterns of local adaptation (see [48]).

#### Migration

Although migration may seem a more specialized type of movement compared to foraging and dispersal, migration is a truly wide-spread phenomenon, including many species of diverse taxa, spanning all modes of locomotion. Although it is difficult to estimate the numbers of individuals migrating each year, initial estimates of some species groups suggest that significant portions of more species migrate than previously assumed. For instance, approximately 60 % of European birds are migratory; in European passerine birds travelling between Europe and Africa, this comprises 2.1 billion birds [49]. Despite these impressive numbers, we hardly know which consequences migrations have for the redistribution of nutrients, other organic and inorganic material, other organisms, seeds, and propagules, or whether and how strongly migrants interact with processes in the various places they visit. Consequences of migrations have only been investigated in some economically important or charismatic species offering useful insights: For example, snow geese (*Anser caerulescens atlanticus*) provide allochthonous resources to Arctic foxes (*Vulpes lagopus*), i.e., geese subsidize a consumer (fox) population, which results in a clear reproductive response by the foxes [50]. Migratory animals can also form enormous pulses of herbivory that might even lead to catastrophic, irreversible ecosystem changes [10], or pulses of migration [51], transport pathogens and parasites [52].

Migration has long been recognized for its uniqueness as well as for the challenges it poses to species conservation [53]. Therefore, migrations have been included specifically as a biodiversity component in the World Wildlife Fund’s Global 200 Ecoregions, a spatially explicit tool for prioritizing conservation areas globally [54]. Conservation needs are also acknowledged in the Convention on Migratory Species (CMS) as an international conservation treaty that develops and implements binding agreements for its members focused on mitigating migration obstacles and conserving migratory species [55]. These conservation efforts and others usually recognize that migration is a major ecological process that is necessary for effective conservation of biodiversity and ecosystems.

### A conceptual framework for better integrating movement into biodiversity research

**Figure 2:**
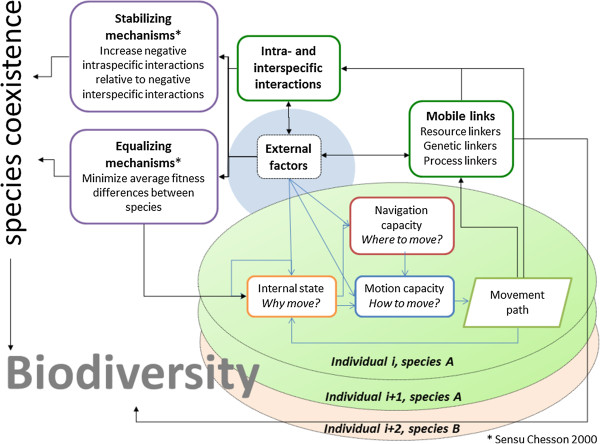
**Integrative conceptual framework for the linkage of movement ecology with biodiversity research.** The movement ecology framework for individuals (after [26]) is linked to the concept of mobile links (see Background information 1) and the concept of equalizing and stabilizing mechanisms for species coexistence (sensu [28], see Background information 2). An individual moves according to its internal state, its navigation capacity and its motion capacity, all of which are affected by external environmental conditions. The resulting movement path feeds back to the internal state. Via the movement path moving animals provide a link between communities and ecosystems that are otherwise separate. Based on what the animals primarily transport and translocate they can be categorized as resource, process and genetic linkers ([27], see Background information 1). Note that the moving individuals may belong to multiple, possibly interacting, species with separate/distinct movement behavior. Effects of mobile links can change external factors (e.g. nutrient levels or cycling) at the connected habitats and ecosystems; they can add new genetic material and species thereby directly impacting biodiversity or they can modify local intra- and interspecific interactions, e.g. through seed transport from source to sink habitats. Intra- and interspecific interactions can also be directly influenced by the specific movement path of individuals, e.g. through active spatial avoidance of competition or predation. Finally, external factors and intra- and interspecific interactions determine the strength and role of stabilizing and equalizing mechanisms in species coexistence (see Background information 2).

Given the different movement types and their potential impact on different levels of biodiversity at different spatiotemporal scales, there is a need for a conceptual framework that allows for a better integration of movement ecology into biodiversity research. A suitable starting point is provided by the conceptual framework for movement ecology related to focal individuals introduced by Nathan et al. [26]. In this framework the authors distinguish between three basic components related to the focal individual, i.e., internal state, motion capacity, and navigation capacity, that are affected by various external factors (summarized as a fourth basic component). The resulting movement path of the individual feeds back to the internal and external components (Figure 2). Extending this framework to biodiversity research requires the addition of key links showing how moving individuals impact biodiversity. To this end, we integrate here two additional existing concepts that should be considered as principal links (Figure 2): First, we integrate the concept of “mobile links” that was developed to describe how moving animals provide a link between communities and ecosystems that otherwise remain separate [27]. Based on what animals primarily transport and translocate between areas, they have been categorized as resource, process, and genetic links (see Background information 1). The mobile links perspective has two components: On the one hand, it shifts the focus from the direct effects of movement on the fitness of the moving species itself (as for example in a metapopulation perspective) towards the view of how movement of one or several species might affect other species through the movement process. On the other hand, it provides a functional perspective, i.e. the movement of individuals is investigated with respect to the effects of the individual movement for a particular question, e.g. seed transport. This provides an important perspective in deciding which elements of the individual movement decisions and resulting paths need to be explicitly resolved.

Secondly, we apply the concept of ‘stabilizing’ and ‘equalizing’ mechanisms that enables a general categorization of mechanisms of species diversity maintenance [28]. In the context of our framework, equalizing mechanisms are effects caused by moving individuals that minimize average fitness differences between species, and stabilizing mechanisms are effects that increase negative intraspecific interactions relative to negative interspecific interactions including intraspecific density-dependent feedback loops ([28], see Background information 2). The concept of stabilizing and equalizing mechanisms extends the conceptual framework for movement ecology by providing a conceptual link to species coexistence mechanisms. While we do not claim that this new integrated conceptual framework (Figure 2) will cover all aspects of how movement can impact the different levels of biodiversity, we see it as a suitable starting point to better organize our ideas and identify gaps in this important and emerging field of integrative research.

#### Background information 1: Mobile links

Organismal movements can provide links between communities and ecosystems, thereby connecting resources, genes and processes among otherwise separated locations. A mobile link is defined ‘as an organism that (i) actively moves across space and time and (ii) thereby connects habitats and influences ecosystem dynamics’ [27]. Typically, the focus of the mobile link concept is on the effect moving individuals have on other species, rather than the direct effect for the species of the moving individuals itself. However, this concept can easily be extended, e.g. in the case of range expansions or source-sink dynamics where moving individuals are key to local or regional species occurrence and persistence. Since we here refer to mobile links from the perspective of the focal process (e.g. transport of nutrients, individuals, genes), this might comprise several moving individuals for which relevant parts of the movement are linked.

Resource linkers transport energy, organic and inorganic material, e.g. nutrients or minerals. Prominent examples include seabirds concentrating nutrients via guano deposits [8], salmon transporting nutrients and energy upstream (and possibly redistributed by scavengers and predators, e.g. [56]), and herbivorous waterbirds carrying nutrients from meadows and pastures into freshwater bodies [9].

Genetic linkers transport mainly ‘genetic material’ into a community, e.g. by transporting genes within seeds, propagules, microbiota or other organisms. Examples include flying foxes on oceanic islands that disperse seeds and pollen of various endemic plant species (e.g. [57]), large herbivores that disperse seeds and improve their germination through endozoochory in Savannah ecosystems (e.g. [58–60]), or a lizard that is the only seed disperser for a specific shrub species and therefore impacts the spatial genetic structure of the plant species (e.g. [61]).

Process linkers engage in some activities that provide new or intensify existing ecological processes. Examples include grazing (e.g. of big mammals or herbivorous birds, which affects nutrient cycling, biomass production, disturbance regimes and consequently plant species composition [10, 62]), or predation (e.g. [63]).

#### Background information 2: Coexistence and the role of equalizing and stabilizing mechanisms

Classical niche theory predicts that in a spatially homogeneous locality, the number of coexisting species should be equal to or less than the number of limiting factors [64–66]. Basically, coexistence through niche differentiation occurs when species are sufficiently different to reduce interspecific competition below intraspecific competition [28, 65, 67]. Interspecific trade-offs such as differential resource use [65, 66], susceptibility to predators [68] or fitness in a temporally variable environment [69] are typically thought to be a requirement for species coexistence in communities at small spatial scales [70].

In general, factors contributing to species coexistence can be categorized into stabilizing and equalizing mechanisms [28]. Stabilizing mechanisms increase negative intraspecific interactions relative to negative interspecific interactions including intraspecific density-dependent feedback loops. They are essential for species coexistence and include traditional mechanisms such as resource partitioning and frequency-dependent predation [28]. By contrast, equalizing mechanisms such as disturbances only contribute to stable coexistence by reducing large average fitness inequalities which might negate the effects of stabilizing mechanisms [28].

We propose here the concepts of equalizing and stabilizing mechanisms as a suitable approach for categorizing the diverse effects of movement on the maintenance of species coexistence (Figure 2). One fundamental effect of the explicit consideration of movement in these concepts is the necessity of giving up the classical mean-field approach, i.e., the assumption of complete mixing of interacting individuals and species (e.g. [71]). Traditional models of population and community dynamics assume well-mixed populations comprising many individuals in which demographic parameters can be defined as functions of overall density [72]. In such models, competing species or predators and prey will encounter each other in proportion to their average abundance over a certain area. As a consequence, reproductive and mortality rates depend on overall population densities whereas the explicit consideration of individual movements in space and time shifts the focus to changing local densities and individual encounters.

### Mobile links and biodiversity

By means of foraging, dispersing, migrating or any other movement activity, mobile linkers can connect genes, resources, and processes among otherwise separate locations [27]. By transporting genetic and other material and facilitating essential processes in communities and ecosystems, such mobile linkers can play a key role for the maintenance or structuring of biodiversity. Direct effects on species and genetic diversity are mainly related to genetic linkers (see Background information 1). For example, 60-80% of all plants and up to 90% of trees in tropical areas are principally dispersed by animals [40, 73]. Disappearance of these animals will seriously impact the spatial distribution of plant species and ultimately biodiversity patterns. The same probably holds for other systems as well, including savanna systems, forests and grasslands [40, 41, 59, 74, 75]. Depending on the type of movement (e.g. foraging, dispersal or migration), the transport of genetic material can cover a broad range of scales. For example, on small scales, seed transport can be restricted to microsites and empty patches within the same habitat (e.g. [76]) or lead to an increased seed rain and colonization of disturbed agricultural areas (e.g. [77]) during foraging activities. On large scales, migratory birds, for example, are responsible for the transport of parasites and pathogens between European and African wetlands or Western Europe and the Arctic [78, 79]. Similarly, migrating birds connect temperate freshwaters in Europe and the Arctic through external or internal seed transport [80]. On smaller scales, pollination by foraging insects or birds connects individuals within or between populations, possibly leading to increased seed set, seedling establishment and plant density (e.g. [81]). In general, gene flow through the action of vectors impacts genetic differentiation and local adaptation across scales (e.g. [44, 48, 61, 82]). Consequently, declining movements of pollinators and dispersers (e.g. mammals, birds) due to landscape fragmentation is expected to result in a dramatic decline of species dependent on these services [77, 83, 84]. Negative impacts on biodiversity can also be expected as a result of increased movements due to human activities. In fact, humans can be considered mobile links in and of themselves, moving other organisms (e.g. invasive) both intentionally (e.g. agricultural products, gardening, pets, assisted migration) and unintentionally (pests, disease-vectors, etc.) (e.g. [85–87]). Biological homogenization is strongly impacted by the movement dynamics of humans and affiliated species, and we must identify and quantify where these movements facilitate or impede the movements of others.

Probably just as important yet more indirect effects of mobile linkers on biodiversity are provided by resource and process links. Local habitats, i.e., local conditions for persistence and coexistence, can be altered significantly by resource links. Examples are freshwaters that are fertilized by nutrient input from waterbirds that forage on pastures and meadows [9, 88], and seabirds that concentrate sea-derived nutrients on terrestrial breeding areas or into adjacent freshwater [89]. Also, anadromous fish (e.g. salmon) that migrate to their natal freshwaters to spawn and die thereby introduce nutrients and energy accumulated in saltwater bodies. Corresponding effects may range from altered vegetation structure to changed (algal) biodiversity [90–92].

Typical process links are provided by foraging animals that impact species interactions and coexistence through selective grazing (e.g. [36, 93], predation (e.g. [38, 94]) or disturbances caused by trampling (e.g. [37]). Grazing also impacts local habitats of migratory herbivorous birds that can change local vegetation structure and composition in an interplay of competition with resident species [10, 62]. Other biodiversity-relevant process links connected to dispersal include spill-over effects of natural enemies in pest control at edges into agricultural systems [47], disease transfer [43, 95, 96], or spatial synchronization in population dynamics affecting metapopulation viability [97].

### Movement and the mediation of coexistence

All three types of mobile links, i.e., genetic, resource and process links, can impact local conditions for intra- and interspecific interactions thus modifying species coexistence. Also movement itself, e.g. the ability of individuals to actively choose the timing and path of movement, allows for avoidance or intensification of species interactions. It can thus impact the degree of mixing within and between species [72] and shape the way in which competition, predation and other species’ interactions affect populations and communities (Figure 2). In either case, the question if and how movement affects species coexistence in a specific system, either directly or through mobile links, depends on its specific impact on intra- and interspecific differences and interactions, i.e. by (i) equalizing, and (ii) stabilizing mechanisms ([28], see Background information 2).

For example, actively avoiding foraging sites with currently high densities of stronger competitors is a typical movement-related behavior in herbivore communities. Such spatiotemporal competition avoidance allows an inferior species to reduce average fitness differences compared to superior species [98] and can thus be termed an ‘equalizing mechanism’ sensu Chesson ([28], see Background information 2). The individuals’ movement responses to the intensity of site-specific competition will affect their individual performance and, in turn, population-level demography [72, 99]. If landscapes include specific regions that are (at least temporarily) under-utilized by the superior species, such competition avoidance can lead to spatial resource partitioning which can cause long-term coexistence (i.e., ‘stabilizing mechanism’ sensu [28], see Background information 2). A good example are grazers in a tall grass African savannah [98], where an inferior competitor (sable antelope) can coexist with superior species (zebra and buffalo) by seasonally moving to regions without zebras or buffalos. Similarly, avoiding areas with higher densities of a joint predator species may counterbalance competitive disadvantages.

Evidently, such mechanisms not only depend on the capacity of individuals to move, but also on the way in which landscape structures facilitate or impede these movements – namely, functional landscape connectivity [100, 101]. For example, numerous studies show that spatial heterogeneity of landscapes in combination with movement-related trade-offs support species coexistence (e.g. [102], see [70] for discussion). Such trade-offs include the relative ability to compete and persist in patches and to move to and colonize new patches [70, 103]. These trade-offs have at least an equalizing effect on average species fitness but they only lead to stability (i.e., are stabilizing factors) if they are linked appropriately to density-dependent feedback loops [28]. At the regional scale, this has been shown for meta-communities, i.e., groups of species that potentially interact and that are spatially segregated into distinct patches connected by dispersal [100, 103]. This patch connection can be attributed to the mobile link concept (‘genetic linkers’) as described above. Interestingly, several studies show that only moderate levels of dispersal/mobility increase local community diversity (e.g. [70, 104–106]). If dispersal rates are too high or too low, negative effects of species mixing and local competition prevail.

Heterogeneity may also arise from spatiotemporal environmental variation, which may promote coexistence through storage effects [28, 107, 108]. Such variation in the environment could be caused by abiotic drivers, such as heterogeneous rainfall patterns, or may be related to mobile resource links (see above). Spatial storage occurs when species show spatially variable responses to the competitive environment that leads to a covariance between the environment and competition. A large body of theoretical studies has demonstrated that such storage effects can drive local coexistence via source-sink dynamics (compare genetic links, Background information 1) under different levels of competition, but to date empirical evidence remains scarce [109]. Here again, movement enables interacting species to make different use of the actual local environmental conditions leading to stabilizing mechanisms of coexistence [28, 108].

Even in homogenous environments, non-homogenous distribution patterns of individuals are another important movement-related mechanism that can stabilize communities of competing species. For example, encounter rates and population dynamics in predator–prey systems can be strongly influenced by the aggregation of individuals, e.g. the active formation of social groups of predators or prey [72]. Such grouping was recently shown to strongly stabilize interactions between lions and wildebeest in the Serengeti ecosystem [110]. Also, the formation of a home range or territory is a stabilizing mechanism that facilitates coexistence by means of spatial resource partitioning. Animals often spend their reproductive period in a region that is small compared with their movement capabilities [72]. In particular, when mobility allows active territorial defense that is more intra- than interspecific, conditions for a stabilizing mechanism are fulfilled [28, 70]. Interestingly, also the specific way in which home range distributions in mammal or bird communities depend on the type of individual foraging movement can influence community response to habitat loss and fragmentation [111–113]. On one side, these findings indicate the importance of individual foraging movement characterized by physiology and behavior for higher levels of biodiversity. On the other side, considering the diversity of movement and foraging strategies present (e.g. [114, 115]), this further illustrates the need to better link movement aspects to projections of biodiversity responses to environmental change.

## Discussion

In light of the ongoing biodiversity crisis, we stress and exemplify here the need for a better integration of movement ecology into biodiversity research. Merging the ‘movement ecology framework for individuals’ [26] with the concepts of ‘mobile links’ [27] and ‘coexistence mechanisms’ [28], we introduce an initial attempt towards a conceptual framework for such integration. More closely connecting research in these largely separate disciplines will not only improve our mechanistic understanding of processes that shape biodiversity across scales but may also contribute to the efficacy of conservation efforts.

### A conceptual starting point

We see our conceptual framework as a starting point to unravel the role of individual movement in biodiversity dynamics. For example, with regard to mobile links, it would be important to better understand whether and to what extent these movements and their different spatiotemporal scales, frequencies and timings have implications for the organization of diversity. Mobile links so far are typically studied on short temporal scales: movements within hours, days or seasons, while measurable effects on biodiversity may require many generations of sometimes long-lived organisms to become detectable. In addition, the cumulative effect of many links from more than one species may be significantly larger than the scale of a single link, which has hampered an explicit recognition of the consequences of animal movements on biodiversity and ecosystem functioning. In this case, multiple mobile linkers from the same species could be defined not as individuals but as groups of individuals following the same functions (i.e. rules, properties). Such groups could be treated as a vector summarizing the behavior of the individual components, including variance and covariance. As such, the approach could also include between-individual variation and predict credibility intervals rather than only the mean. Individuals or groups could also be linked to other mobile links (e.g. other species), creating a ‘mobile chain’ which would be very relevant at community or ecosystem levels. One challenge in this context is that despite rapid technological developments our ability to track multiple individuals over long time periods or even entire life-cycles is still limited. The same still holds largely for our understanding of factors that shape animal movement decisions and patterns (e.g. pathway, distance, duration, frequency, etc.). Some of the open questions may be addressed with newly developed technology and research tools such as geolocators, isotope and genetic markers (e.g. [116, 117]) and the aggregation of such data in online-platforms (e.g. [118]). However, weight and lifetime of currently available tracking devices are still non-permissive for many animals or extended periods of time. Low orbit or airplace mounted active antenna arrays, as proposed by the ICARUS project [119, 120], may address many of these size and weight challenges for tracking small animals in the future.

However, another challenge is posed by the context-dependence of the potential effect the mobile link may have. Whether the transport of, e.g., nutrients or seeds by moving animals is relevant for biodiversity and ecosystem functions not only depends on the specific link created but also on the specificities of the communities and ecosystems connected. For example, waterbirds might disperse freshwater snails, but dispersal success strongly depends on the birds reaching another body of water within retention times (e.g. [80]). Similarly, in the case of frugivorous birds the effect of seed transport strongly depends on the local plant community composition (e.g. [74]). Important to note here is that mobile linkers interact with other species and the physical environment at a specific place and the outcomes of these interactions will also determine the fate of the linkers, including their propensity to continue moving.

Using the framework of equalizing and stabilizing mechanisms may help unravel the effects mobile links can have on local communities. Crucial questions to ask would include the following: (Under which circumstances) can we expect the mobile link to reduce average fitness differences between species? (Under which circumstances) can mobile links even cause non-linear, density-dependent feedback? The context dependency of these dynamics can be nicely illustrated by the presence of floaters in bird populations. Floaters are adult non-reproducing individuals in a population. While they probably do not reduce average fitness differences between species they may become a crucial population reserve for filling empty territories, e.g. when breeding dispersal or mortality vacates previously occupied territories [72]. On rare occasions this may happen simultaneously to several territories, e.g. after intense or large-scale disturbances. However, on such occasions mobile floaters can form a non-linear, density-dependent buffering mechanism when populations face risk of extinction [121] and thus also form a stabilizing mechanism [28]. By contrast, if the population is at a higher density, floaters are merely competitors of the territory-holders and their offspring and as such impact the population growth rate.

Apart from mobile links, mobility has a clear impact on species coexistence through the mediation of stabilizing and equalizing mechanisms. Identifying simple movement rules might already reveal some stabilizing mechanisms that can explain species coexistence, such as prey switching in systems with one or more predators and several prey species (i.e., predators focusing on the currently more abundant prey species, e.g. [122]) or other non-linear density-dependent feedback loops, where negative interactions are strongly reduced at low densities (e.g. [123]). However, only a full and more detailed consideration of movement will enable a better understanding of more spatially explicit and thus more realistic stabilizing mechanisms, including the prevalence of mixed strategies like partial migration (e.g. [124]). For instance, in a simple food web consisting of two competitors and a single predator, competition-mobility trade-offs will induce coexistence when dominant competitors suffer from density-dependent control (individual or population growth rate) by predation, while subordinate competitors are less vulnerable to predation by more successful spatiotemporal predator avoidance [70]. It can be expected that in more complex systems and food webs movement-related mechanisms for coexistence are even more complex. In particular, when landscapes are altered by land use or other human activities, identifying effects on movement-related equalizing or stabilizing mechanisms will be highly challenging. However, detecting such effects will be crucial to better inform conservation management [125, 126].

### Adding a methodological perspective

Linking movement ecology with biodiversity research using the framework introduced here will undoubtedly still pose challenges originating from mismatches in the focal scales of the two fields (Figure 1), the immense stochasticity and variability emerging from individual movement, and the need to identify means of reducing complexity when upscaling from individuals to ecosystems and increasing the number of species for which movement data are collected. We see three non-exclusive approaches to overcome some of these challenges in order to advance the urgently required integration of these research fields: (i) landscape-level experiments, (ii) individual-based modelling, and (iii) landscape genetics.

#### Landscape level experiments

A major reason for such scant experimental proof of specific movement effects on biodiversity might be the complexity of animal movement and especially the complexity of the outcomes of animal-landscape interactions. On top of that, there is a need to quantify the effects of mobile links on biodiversity components or ecosystem features. Here, experimental setups may be imperative. One research area on which experimental field work has successfully been accomplished is the effect of animal-mediated dispersal (epi- and endozoochorous) on the diversity of local communities (i.e. ‘genetic linkers’, see Background information 1). For example, experimental studies demonstrate the effect of surrounding vegetation on seed rain by frugivorous birds in pine plantations [127], the effect of the transport of a large array of plant species within and between nature reserves by domesticated ungulates [128], and effects of animal corridor movement on plant diversity in patches that are connected to conservation areas [129]. Other attempts to address such relations can be seen in the work of Green & Figurola [130] on the diversity of aquatic invertebrates with relation to dispersal by birds. Experiments are, however, typically designed to investigate processes at small (plot) scales, and the link to diversity patterns at larger scales in consideration of (animal) movement remains largely unexplored. Therefore, there is a strong need for landscape-level experiments that on the one hand systematically change landscape features (e.g. barriers or corridors, [131]) or management (e.g. type, timing or intensity of land use and disturbances) and on the other hand monitor resulting movement changes across different taxa as well as short and medium term impacts on biodiversity and ecosystem services. Such experiments become feasible due to the development of automated tracking systems that can cover large areas and large numbers of animals simultaneously [119, 120], but suffer inevitably from design problems in the sense of proper replication.

#### Individual-based modelling

Movement ecology is a typical bottom-up approach and aims at improving mechanistic understanding of movement processes by focusing on the individual level [132]. Therefore, spatially-explicit, individual-based modelling is a suitable approach to integrate individual movement into an eco-evolutionary simulation framework to derive its consequences at higher organizational levels [133, 134]. For instance, linking individual-based spatial models of species coexistence with novel movement data and experiments will help identify equalizing or stabilizing mechanisms by identifying long-term consequences of different movement aspects at the individual level on community performance. This includes studying the relevance of individual differences in movement (e.g. due to different phenotypes, past experience, individual condition, or even ‘personalities’; [72]). Distinguishing the effects of individual movement that merely reduce average fitness differences between species from effects that intensify intra- versus interspecific competition leading to non-linearities that stabilize coexistence will help to bridge the gap between behavior, landscape ecology, climate change ecology, and biodiversity.

The complexity of a large number of interacting individuals has so far limited direct process-based and bottom-up simulations of biodiversity consequences (but see [112, 135]). Revilla & Wiegand [136] provide a representation of how the movement-ecology paradigm can be linked to individual fitness, ultimately to predict demographic rates at the population level. In a case study involving the Iberian Lynx (*Lynx pardinus*), they showed that individual movement behavior and survival interact dynamically with profound effects on local population and meta-population dynamics. Extending this framework to the (meta-)community level can provide a clear mechanistic representation of how the movement of organisms affects community dynamics [137] and vice versa how intra- and interspecific interactions affect movement and fitness of individuals [138, 139]. The complex feedback between different processes and entities involving genetic, resource and process links can probably only be systematically explored in future modeling approaches that succeed in finding a convincing balance between inherent complexity and necessary simplification and information aggregation. Clearly, the success of individual-based models to support such advances will also largely depend on the capacity to develop effective methods for upscaling, as small-scale modeling approaches cannot be used to generate predictions over large areas or large number of species without risk of bias [19]. This will be particularly relevant in situations where multiple species need to be considered that are moving at separate characteristic scales.

#### Landscape genetics

In addition to individual movement data, genetic data can provide detailed information on certain movement events and its consequences, particularly with respect to mating movements and successful dispersal (e.g., genetic exchange within and among populations). Specifically, one main focus of the young field of landscape genetics is to quantify how the quality and configuration of matrix land cover types influence genetic links and resulting genetic patterns [140, 141]. The number of studies that use genetic data to understand the effects of environmental heterogeneity on individual movement, dispersal success and subsequent reproduction is growing exponentially (see [142]), and landscape genetics has already begun to incorporate individual movement as a major mechanism explaining spatial genetic patterns (e.g. [4]). Another relatively young field called community genetics could also benefit greatly from the movement ecology paradigm for the purposes of explaining and predicting observed genetic patterns as a consequence of individual movements. Community genetics centers on the population genetics of ecological communities (i.e., multiple species) and its interplay with the evolutionary dynamics of these communities [143, 144]. Amalgamating these three fields – movement ecology, landscape genetics, and community genetics – could create unprecedented opportunities for understanding the exact impacts of genetic links on spatiotemporal biodiversity dynamics across levels and scales.

## Conclusion

We have only recently begun to understand the mechanisms leading to individual variation of movement and the impact of movements on processes that shape different levels of biodiversity at different spatiotemporal scales. Clearly, since movement is fundamental for predicting human impact on biodiversity, e.g. in the context of changes in landscape configuration, habitat deterioration and climate change, we advocate that a more integrated approach based on a joint conceptual framework is essential. While behavioral adaptation in movement patterns may buffer negative effects of habitat or climate changes on communities, it will be important to distinguish whether these effects merely slow down species loss or whether they have a longer-lasting or even permanent stabilizing effect. Also, it will be important to identify scenarios where movement potentially intensifies negative biodiversity effects of human activities. Classical (behavioral) ecological approaches at different spatial scales, extended by means of sophisticated geolocation, isotopic and molecular profiling, large-scale experiments and advanced spatially explicit modeling approaches, are essential for understanding variation in movement, its translation to statistical properties and the processes structuring diversity. Recent technological advancements certainly allow the collection, processing and analysis of more and better data on movement patterns, but a key challenge now is to make full use of such data with regard to better understanding and predicting biodiversity dynamics. This will require continuous refinement of the integrative conceptual framework presented here spanning the spectrum from drivers of individual movement to the variety of biodiversity levels across spatiotemporal scales.
